# Temperate seaweeds *Himanthalia elongata* and *Fucus vesiculosus* significantly reduce rumen methane emissions *in vitro* due to their high phlorotannin content

**DOI:** 10.1002/jsfa.70016

**Published:** 2025-07-02

**Authors:** Kayley Barnes, Maria Hayes, Farley Miller, David Yanez‐Ruiz, Elisabet Jimenez, Lucy Dillon, Emmet Campbell, Philip McCarron, James Pickup, Sharon Huws, Katerina Theodoridou

**Affiliations:** ^1^ School of Biological Sciences/Institute for Global Food Security Queen's University Belfast Belfast UK; ^2^ Food BioSciences Department, Teagasc Food Research Centre Dublin Ireland; ^3^ Estación Experimental del Zaidín, Consejo Superior de Investigaciones Científicas (CSIC) Granada Spain

**Keywords:** seaweed, methane, ruminant, rumen, *Himanthalia elongata*, *Fucus vesiculosus*, *Asparagopsis* spp.

## Abstract

**BACKGROUND:**

Global food insecurity and the fact that food production contributes around 30% of greenhouse gas (GHG) emissions is a major planetary challenge. Ruminant products are widely consumed since they are macro‐ and micronutrient dense; however, ruminants produce enteric methane (CH_4_), a potent GHG. Feeding seaweeds, such as *Asparagopsis* spp., to ruminants reduces enteric CH_4_ emissions. This study investigates the CH_4_ mitigation potential of seaweeds, including *Alaria esculenta* (AE), *Ascophyllum nodosum* (AN), *Asparagopsis taxiformis* (AT; positive control), *Chondrus crispus* (CC), *Fucus vesiculosus* (FV), *Himanthalia elongata* (HE) and two seaweed‐derived extracts – *Himanthalia elongata* (XHE), and *Chondrus crispus* (XCC) – on rumen fermentation (CH_4_, ammonia (NH_3_), volatile fatty acids (VFA) and pH) at three timepoints (4, 24 and 48 h). The contents of volatile organic compounds (VOC) and phlorotannin (PT) of the seaweeds were also investigated for insight into the mode of action.

**RESULTS:**

As expected, AT consistently reduced CH_4_ production in comparison to the respective negative grass silage controls (~93.3%) and other tested seaweeds (*P* < 0.05) at all timepoints. At 4 h AN, FV, XCC and XHE elicited a reduction in CH_4_ of 2.0%, 3.0%, 40.9% and 31.1%, respectively, over the negative controls. XHE was the only tested seaweed to reduce CH_4_ production (4.9%) at the 24 h timepoint. At 48 h FV, CC, HE, XCC and XHE showed reductions of 14.4%, 2.9%, 1.9%, 2.8% and 42.8%, respectively, over the negative controls.

**CONCLUSION:**

As a consequence of their high PT content, XHE and FV show promise for GHG mitigation in ruminants, thereby aiding ruminant food security. © 2025 The Author(s). *Journal of the Science of Food and Agriculture* published by John Wiley & Sons Ltd on behalf of Society of Chemical Industry.

## INTRODUCTION

The livestock sector plays an important role in food security; however, transformation to sustainable livestock production practices is vital for planetary health.^1^ Ruminants produce methane (CH_4_) as a metabolic end‐product of enteric fermentation within the rumen and is the largest source of greenhouse gas emissions in agriculture, accounting for 6% of total greenhouse gas emissions globally.^2^ CH_4_ is under increased scrutiny due to its heightened global warming potency compared with carbon dioxide (CO_2_), being 28 times more effective at trapping heat over a 100‐year period,^3^ although CH_4_ has a shorter half‐life of approximately 12 years.^4^ This suggests that reducing livestock CH_4_ emissions may result in a more instant effect in climate change. In 2021, 149 countries pledged to reduce CH_4_ emissions by 30% by 2030.^5^ As a result, research intensity on finding reduction solutions to enteric CH_4_ production from ruminants has heightened, with the use of feed additives or alternative feed ingredients being an attractive strategy due to the ease of applicability, cost‐effectiveness and instant results over other options (e.g., genetics, vaccines).^6^, ^7^

Macroalgae, commonly known as seaweed, have shown potential as a CH_4_ inhibitor in ruminants.^8^ Seaweeds represent a large domain of aquatic plants, separated into three main taxa: Chlorophyta (green), Phaeophyceae (brown) and Rhodophyta (red).^9^ Across the taxonomic groups, over 3000 bioactive secondary metabolites have been identified, with a diverse range of benefits to human and animal health.^10^ Some of these secondary metabolites such as halogenated compounds and phlorotannins (PTs) are responsible for anti‐methanogenic properties,^11^, ^12^ while benefits to end‐product quality can arise from enhancing various other nutrients such as minerals, protein and unsaturated fatty acid content.^12^, ^13^, ^14^, ^15^ The potential of seaweeds as an enteric CH_4_ mitigation method is largely related to the tropical red *Asparagopsis* spp. When provided to ruminant diets at inclusions >1%, reductions of ~98% have been reported *in vitro* ^16^ and ~ 80% *in vivo*.^17^, ^18^ The causative bioactive is the halogenic compound bromoform (CHBr_3_), which is thought to interfere with methanogenesis by blocking the action of key enzymes in the Wolfe cycle (reduction of CO_2_ to CH_4_ in the general reaction CO_2_ + 4H_2_ to CH_4_ + 2H_2_O by rumen hydrogenotrophic methanogenic archaea).^19^, ^20^ While *Asparagopsis* spp. shows promise as a CH_4_‐mitigating feed additive, there is still research required to assess the effect of CHBr_3_ on animal health and end‐product quality. For example, Muizelaar *et al*.^21^ reported abnormalities of the rumen wall papillae from the high *Asparagopsis taxiformis* (AT) treatment group (333 g dry matter (DM) AT inclusion) and a reduced voluntary feed intake in dairy cows at all AT inclusion levels (67–333 g DM). In the same study, CHBr_3_ was detected in milk during the first 9 days and urine for the first 10 days of the 22‐day feeding period, whereas other studies have not detected CHBr_3_ compounds in the animal tissues or milk from *Asparagopsis* spp. provision.^18^, ^22^, ^23^ However, it should be noted that the inclusion level at which AT was provided varied across studies, yet they shared the commonality of significant reductions in CH_4_ emissions.

CHBr_3_ is predominantly found in red and green seaweed species, while PTs are principally found in brown species PTs are polymers of phloroglucinol (1,3,5‐trihydroxybenzene) units and constitute most of the phenols present in brown algae;^24^ while being analogues to terrestrially found condensed tannins (CT), they are structurally different.^25^ The mode of action of PTs is speculated to be similar to that of CTs, especially regarding the effects on enhancing protein efficiency in ruminants through increasing bypass protein provision^26^; CT provision at levels between 2% and 4% of dietary dry matter has positive effects in ruminants by increasing protein metabolism,^27^ and reducing bloat and enteric CH_4_ emissions.^28^, ^29^, ^30^ It is believed that they primarily interfere with the degradability of neutral detergent fibre (NDF) by inhibiting the action of cellulolytic bacteria,^26^, ^31^ decreasing the amount of H_2_ available for methanogens;^32^, ^33^ they also have antiprotozoal activity.^33^, ^34^

The PT level varies both within and across brown seaweed species, due to biotic and abiotic factors, and can range from 0.5% to 20% of the algal DW,^8^ with some species having notably higher concentrations: *Himanthalia elongata*, *Fucus vesiculosus* and *Ascophyllum nodosum* commonly have 321, 207 and 107 mg g^−1^ DM, respectively.^35^ Roskam *et al*.^35^ investigated the effect of the aforementioned species on rumen fermentation *in vitro* at a 1% inclusion and found no effect on absolute CH_4_ output or fermentation patterns, suggesting that, while PT content was high, the inclusion rate was too low to elicit an effect. Most other *in vitro* studies that investigate temperate species use inclusion rates < 5%,^36^ which raises cost, animal health and iodine content concerns if provided at the same rate *in vivo*.^20^, ^37^ High dietary seaweed inclusion *in vivo* often coexists with adverse effects such as decreased dry matter intake and feed efficiency (average daily gain:feed intake ratio).^14^, ^38^ It has been theorised that extracting the bioactive compounds from seaweeds would circumvent the iodine and heavy metal issue, allowing for greater dietary inclusion and thus greater CH_4_ mitigation.^26^

The aim of the present study was to investigate the volatile compounds, PT quantity and the subsequent impacts on rumen fermentation characteristics (CH_4_, ammonia, pH and volatile fatty acids (VFA) production) *in vitro* of six seaweed species (*Alaria esculenta*, *Ascophyllum nodosum*, *Asparagopsis taxiformis* (as a positive control), C*hondrus crispus*, *Fucus vesiculosus* and *Himanthalia elongata*) and two seaweed extracts (*Himanthalia elongata* and *Chondrus crispus*) at a 4% inclusion rate. The inclusion rate of 4% was selected based on the upper feeding rate that could be provided *in vivo*, based on the legal limits for minerals and trace elements found in seaweed species and palatability issues observed *in vivo* when included at above 4% dry matter intake.^39^ These seaweed species were chosen based on biomass availability, biochemical composition and geographical location, the latter to reduce the costs and carbon implications of shipping linked to the additional energy expenditure, should these options be implemented on farm in the future. As such, discovering a temperate seaweed with an anti‐methanogenic response in ruminants would be of global significance, as this would reduce the need for terrestrial innovations for the cultivation of red species, while reducing the wider life cycle impact of providing seaweed to ruminants in temperate regions.

## MATERIALS AND METHODS

### Seaweed samples

A total of five whole temperate seaweed samples (*Alaria esculenta*, *Ascophyllum nodosum*, *Chondrus crispus*, *Fucus vesiculosus* and *Himanthalia elongata*) and two seaweed extracts (*C. crispus* and *H. elongata*) were evaluated against a grass silage negative control and *Asparagopsis taxiformis* positive control. The seaweed form, pigmentation, supplier, origin, harvest season and year are outlined in Table 1. The silage was frozen at −20 °C and freeze‐dried (L‐series bench top, Lablyo, York, UK) for 3 days, milled to 1 mm and stored at 4 °C before being used as the base substrate in the experiment. The grass silage used was prepared in bulk and stored in a homogeneous mix prior to the first *in vitro* experiment to ensure consistency.

**Table 1:** Abbreviations used, seaweed form, pigmentation, supplier, origin, harvest season and year the seaweeds were tested *in vitro*, and the base substrate of grass silage

Substrate	ID	Form	Form	Pigmentation	Supplier	Origin	Harvest season and year
Grass silage	CON	Freeze‐dried and milled (1 mm)	—	—	Agri‐Food and Biosciences. Northern Ireland	Northern Ireland	Summer 2022
*Alaria esculenta*	AE	Whole, air‐dried	Whole	Brown	Teagasc	Cork, Ireland	Summer 2020
*Chondrus crispus*	CC	Whole, air‐dried	Whole	Red	Teagasc	West Coast, Ireland	Summer 2021
*Fucus vesiculosus*	FV	Dried and milled (1 mm), air‐dried	Whole	Brown	Teagasc	West Coast, Ireland	Summer 2020
*Himanthalia elongata*	HE	Dried and milled (1 mm), feed grade, low‐temperature dried (<28 °C)	Whole	Brown	Teagasc	Mayo, Ireland	Summer 2020
*Ascophyllum nodosum*	AN	Whole	Whole	Brown	Ocean Harvest Technology Ltd	Ireland	Summer 2021
*Asparagopsis taxiformis*	AT	Whole, freeze‐dried	Whole	Red	Teagasc	Azores, Portugal	June, 2021
*Chondrus crispus*	XCC	Extract, freeze‐dried, milled.	Extract	Red	Teagasc	Ireland	Extract generated December 2020
*Himanthalia elongata*	XHE	Extract, freeze‐dried, milled.	Extract	Brown	Teagasc	Ireland	Extract generated December 2020

### Generation of seaweed extracts

In addition to testing whole seaweeds, we also wanted to test the possibility of using seaweed extracts that might allow for inclusion levels to be increased due to deceased iodine and heavy metal concentrations, which were removed in the extraction process. The *Himanthalia elongata* extract (XHE) and *Chondrus crispus* extract (XCC) hydrolysate were generated from seaweed using xylanase powder (from *Aspergillus oryzae*, >2500 units g^−1^; Merck, Dublin, Ireland). 100 g of the respective dried and milled seaweed was placed in 1000 mL ddH_2_O in a bioreactor. The reaction was maintained at pH 3.80 and 70 °C using 1 mol L^−1^ HCl and the bioreactor temperature probe controller. The hydrolysates were generated using a New Brunswick (Cambridge, UK) 1.5 L bioreactor with temperature and pH control. The substrates were held in the set bioreactor conditions for 3 h before the hydrolysate was heat‐deactivated by heating the mixture in a water bath to 95 °C for 15 min to deactivate the enzyme. The mixture was cooled to room temperature, frozen at −20 °C and subsequently freeze‐dried.

### Seaweed chemical composition, polyphenol and PT extraction

The protein content of seaweed samples was assessed using an FP628 protein analyser (LECO, St Joseph, MI, USA) and the Dumas method (AOAC Method 992.15, 1990). Recommended N conversion factors were used to determine protein contents. The nitrogen‐to‐protein conversion factor was determined for each seaweed as described by Biancarosa *et al*.^40^ Lipids were quantified using the Smart System 5 microwave moisture drying oven and NMR Smart Trac rapid fat analyser (CEM, Matthews, NC, USA; AOAC Official Methods 985.14 and 985.26, 1990). Ash was determined using a muffle furnace (Carbolite, Hope, UK) overnight at 600 °C (method ISO 2171).

Polyphenols were quantified after extraction from test samples.^41^ Phloroglucinol (Merck) was used as the standard to formulate a standard curve and results were expressed as percent phloroglucinol equivalents (PGE). Samples were assayed in triplicate, three times (*n* = 9), with the exception of AT and XHE, which were assayed in triplicate, twice (*n* = 6). The method of Zhang *et al*.^41^ was used, with 20 μL of each sample solution and the serial standard solution pipetted on a 96‐well microplate. To this, 100 μL of Folin–Ciocâlteu reagent was added, the plate was mixed well and, after a period of 5 min, 80 μL of 7.5% sodium carbonate solution was added to each test well. The 96‐well plate was covered and left for 2 h in the dark at room temperature. The absorbance of each well (*λ* = 620 nm) was measured using a spectrophotometric microplate reader. Samples were mixed on a shaking plate at 200 rpm prior to analysis and values recorded for calculations as described previously.

The PTs were extracted according to the method of Lopes *et al*.^42^ Briefly, 5 g of each sample were extracted with 100 mL hexane. The sample was stirred at 400 rpm for 1 h and lipid was removed (Isotemp, Thermo Fisher Scientific, Waltham, MA, USA). The defatted feed samples were air‐dried overnight. Subsequently, 100 mL acetone–dH_2_O (70:30, v/v) was applied to the defatted sample and this was then extracted four times at 700 rpm for 1 h. Acetone was separated from the organic fractions using rotary vacuum evaporation with an evaporator (model no. 1000243991, Mason Technology, Buchi, Switzerland). Organic fractions were pooled and dried under nitrogen at 30 °C for 3 h. To purify PTs, the sample was weighed and mixed with an equal weight of cellulose; two volumes of methanol were added per 30 mL (w/v) and washed with toluene to remove pigments. PTs were released from the cellulose using acetone–dH_2_O (70:30, v/v). All PT fractions were subsequently dried under vacuum and freeze‐dried.

### Solid‐phase micro‐extraction

The solid‐phase micro‐extraction (SPME) method was used to identify key differences in the content of volatile compounds across the tested seaweed species to help infer the potential mode of action. The SPME fibre used was made from divinylbenzene/carboxen/polydimethylsiloxane (1 cm 50/30 μm DVB/CAAR/PDMS stable flex/ss) and the SPME liner (2637501) was purchased from SUPELCO (Bellefonte, PA, USA). SPME headspace vials (20 mL screw neck, vial ND18 clear 75.5 × 22.5 mm round bottom, SU860097) and PTFE screw caps (SU860101) were purchased from Supelco.

Prior to sample analysis, the SPME fibre was conditioned at 220 °C for 1 h. Test samples (1 g) were weighed into 20 mL SPME vials in duplicate. The vials were immediately sealed with PTFE/silicon liner screw caps. After sealing, vials were thermally equilibrated (6 min) on an incubator and the SPME fibre was inserted into the headspace of the sample vial (depth of 70 mm, 5 min, 75 °C). Following extraction, SPME fibres were inserted into a gas chromatograph (2 min at 270 °C). A blank vial was tested to check for any carryover after every six samples.

Samples were analysed using an Agilent 7890/7010 gas chromatography–mass spectrometry (GC‐MS) triple‐quadrupole instrument (Agilent Technologies, Inc., Santa Clara, CA, USA). Full‐scan mass spectra were acquired over a mass range of 30–550 Da (5.2 scans s^−1^) in splitless mode. The injector, ion source and transfer line temperatures were 250, 230 and 280 °C, respectively. Initial oven temperature (50 °C for 1 min) was increased (170 °C, 10 °C min^−1^) and held for 2 min. Final temperature (280 °C, 30 °C min^−1^) was held for 1 min. Helium carrier gas was 1.0 mL min^−1^. A DB‐5 MS capillary column (60 m length × 0.25 mm internal diameter × 0.25 μm film thickness) was used. Agilent Unknowns Analysis (Version 10.1, Build 10.1.733.0) with NIST 1.4 Library software were used for compound identification. Probability match was set to 80% and chemical compounds were removed if not present in both replicates. Cytoscape was used to visualise the data.

### *In vitro* incubations

Rumen fermentation *in vitro* experiments were conducted to identify the effects of different seaweeds on methane (CH_4_), ammonia (NH_3_), VFA production and pH at a 4% inclusion rate. Samples of the grass silage and/or treatment seaweed were used as substrates for the batch culture of rumen microorganisms. Rumen contents were collected from six cows at random (unknown breed, age, sex and diet) post‐slaughter at the abattoir, stored in thermos flasks, and delivered to Queen's University Belfast within an hour of collection on the start day of the incubation. The rumen contents from each cow were kept separate throughout to make six biological replicates per treatment and timepoint. Rumen contents were strained through four layers of cheesecloth, pH tested (below pH 5.8 deemed unsuitable for use) and mixed with Van Soest buffer solution^43^ in a 50:50 ratio (v/v) at 39 °C. The buffer jars were autoclaved for 15 min at 121 °C prior to mixing with the rumen fluid, with a final pH of 6.8. Throughout the experimental set‐up the rumen fluid, buffer and incubation vessels were kept under a continuous stream of CO_2_ to maintain anaerobic conditions.

For incubation vessels, 100 mL Wheaton glass serum bottles were used. Six replicates of the treatment seaweeds and grass silage were weighed (total weight 1.60 g) and included in the serum jars. The 60 mL volume of buffered rumen fluid was added anaerobically under a continuous stream of CO_2_ using a Prospenser Plus 10–60 mL dispenser (Sartorius, Göttingen, Germany) connected to a stream of CO_2_ via the recirculation valve. Bottles were sealed with butyl rubber stoppers and aluminium caps, and incubated at 39 °C and 80 rpm in a shaker incubator.

Three incubation periods were investigated – 4, 24 and 48 h – to account for differences in the microbial breakdown of feedstuffs and digestion of different fractions. The 4 h timepoint accounts for the fermentation of rapidly fermentable substrates, with the early gas production and VFA profile providing an indication of fermentation rate and microbial activity. The 24 h timepoint captures the mid‐phase fermentation and accounts for the utilisation of most of the soluble and fermentable fibre fractions, while the 48 h timepoint captures complete fermentation of fibre fractions. The closed‐batch system is deemed inappropriate for longer durations due to the build‐up of pressure within the incubation vessel, with the cumulative gas production inhibiting microbial activity.

Gas was collected from the bottle headspace for CH_4_ analysis at the three outlined timepoints. An Agani 18 g × 1.5″ needle (Terumo, Somerset, NJ, USA), gas stopper and 12 mL syringe were used to take 10 mL of gas per serum bottle, which was transferred to a vacuumed 12 mL Exetainer flat‐bottom, evacuated vial (Labco, Lampeter, UK). The CH_4_ concentration (CH_4_ per millilitre of sample) was determined by GC using an HP Hewlett 5890, Packard series II chromatograph (Waldbronn, Germany) and an HP‐Innovax column (25 mm length × 0.2 mm internal diameter × 0.2 um film thickness; Supelco, Madrid, Spain). The carrier gas was nitrogen set at a flow rate of 1 mL min^−1^. Injector and detector temperatures were 250 and 275 °C, respectively. The split ratio was 1:80, and the oven temperature was set at an isothermic program of *T* = 110 °C. A sample of 0.1 mL of the test gas was injected using a 1 mL SampleLock syringe (Hamilton Co., Reno, NV, USA). The CH_4_ was quantified against a standard curve, developed by manual injection of six different amounts of pure CH_4_ in triplicate.

The pH was recorded immediately after the bottle was opened using a pH probe (LE pH electrode LE438‐IP67, Mettler Toledo, Greifensee, Switzerland). Two 1.5 mL samples were taken from each bottle and centrifuged (4 °C, 20 000 × *g*, 10 min). The supernatant was removed and transferred to 2 mL microcentrifuge tubes and stored at −20 °C until required for further investigation of NH_3_ and VFA contents in the medium. NH_3_ quantification in the samples was done according to the Chaney and Marbach technique,^44^ wherein the absorbance in the samples was measured using a CLARIOstar Plus microplate reader (BMG LABTECH, Ortenberg, Germany) at 630 nm. VFAs and alcohols in the samples were measured following ethanol/water extraction via GC analysis, using a Varian Star 3400 CX gas chromatograph (Varian Medical Systems, Palo Alto, CA, USA), equipped with a Zebron ZB‐FFAP column (25 m length × 0.53 mm internal diameter × 1.0 mm film thickness; Phenomenex, Torrance, CA, USA) for on‐column injection with flame ionization detection. Nitrogen was used as the carrier gas with a flow rate of 1 mL min^−1^.

Due to the quantity of incubation bottles required (*n* = 6 per treatment per timepoint), the seaweeds were tested across four incubations using rumen fluid from different cows for each experiment. For comparability, the results from the incubations are expressed as percent change from their respective negative controls, with the exception of pH.

### Statistical analysis

All statistical analyses were conducted using R (version 3.4.1.). Datasets were checked for homogeneity of variance and normality using the Levenes and Shapiro–Wilk test, respectively. Datasets meeting these assumptions were analysed using analysis of variance with Bonferroni post hoc adjustment. Datasets that did not meet the assumption of normality were analysed using non‐parametric methods, specifically the Kruskal–Wallis test followed by Dunn's post hoc test with Bonferroni adjustment.

### Script availability

To create count files for data visualisation from standardised GC‐MS/MS SPME output files, data were manipulated using a combination of Bash and Python code, available at: https://github.com/KayleyBarnes/In_vitro_VOC_code/.

## RESULTS

### Seaweed chemical characteristics

The lipid content of *Ascophyllum nodosum* (AN) (3.49%) was significantly higher than that of *Himanthalia elongata* (HE) (0.41%) and XCC (0.36%) (*P* < 0.05; Table 2). A significant difference was observed between *Alaria esculenta* (AE) and AN (*P* < 0.05; Table 2) for ash content. The phenolic content was significantly higher in XCC compared to AT (*P* < 0.05; Table 2). The phenolic content of XCC could be related to the presence of antioxidants like daidzein or genistein, resulting in increased PGE percent for both XCC and *Chondrus crispus* (CC). While not significant, out of the brown seaweeds AN and HE had the highest total phenolic content, whereas XHE had the greatest quantity of PTs (PGE %) (Table 2).

**Table 2:** The carbohydrate, protein, lipid and ash (%) and the total phenolics (phloroglucinol equivalents, PGE %) and phlorotannin (PGE %) content of the seaweeds tested *in vitro*

	AE	AN	AT	CC	FV	HE	XCC	XHE	*P*‐ value
Carbohydrate (%)	50.25 ± 4.02	61.65 ± 3.66	47.73 ± 12.05	57.67 ± 8.08	59.54 ± 3.07	67.49 ± 6.20	57.45 ± 3.60	64.79 ± 8.79	Ns
Protein (%)	13.73 ± 5.49	7.22a ± 0.38	15.87 ± 6.71	13.52 ± 1.48	11.6b ± 0.34	4.73c ± 0.05	9.7a ± 0.43	8.70 ± 0.86	<0.05
Lipid (%)	0.65 ± 0.04	3.59a ± 1.16	0.57 ± 0.09	0.49 ± 0.39	1.89 ± 1.12	0.41b ± 0.11	0.36b ± 0.09	1.33 ± 0.79	<0.05
Ash (%)	30.74a ± 0.00	19.33b ± 0.80	32.43 ± 22.2	24.11 ± 0.86	20.54 ± 0.93	19.19 ± 6.56	20.65 ± 1.26	25.18 ± 8.80	<0.05
Total phenolics (PGE %)	4.63 ± 1.43	8.21 ± 3.70	5.10a ± 2.29	6.05 ± 0.85	6.44 ± 1.75	8.17 ± 0.63	11.82b ± 3.05	6.09 ± 1.32	<0.05
Phlorotannin (PGE %)	Nd	2.10 ± 0.60	Nd	Nd	3.57 ± 3.62	4.26 ± 0.70	Nd	7.45 ± 0.26	Ns

Letters after entries indicate significant differences across treatments (*P* < 0.05). Nd, not described; Ns, not significant; AE, *Alaria esculenta*; AN, *Ascophyllum nodosum*; AT, *Asparagopsis taxiformis*; CC, *Chondrus crispus*; FV, *Fucus vesiculosus*; HE, *Himanthalia elongata*; XCC, *Chondrus crispus* extract; *X*HE, *Himanthalia elongata* extract.

**Figure 1:**
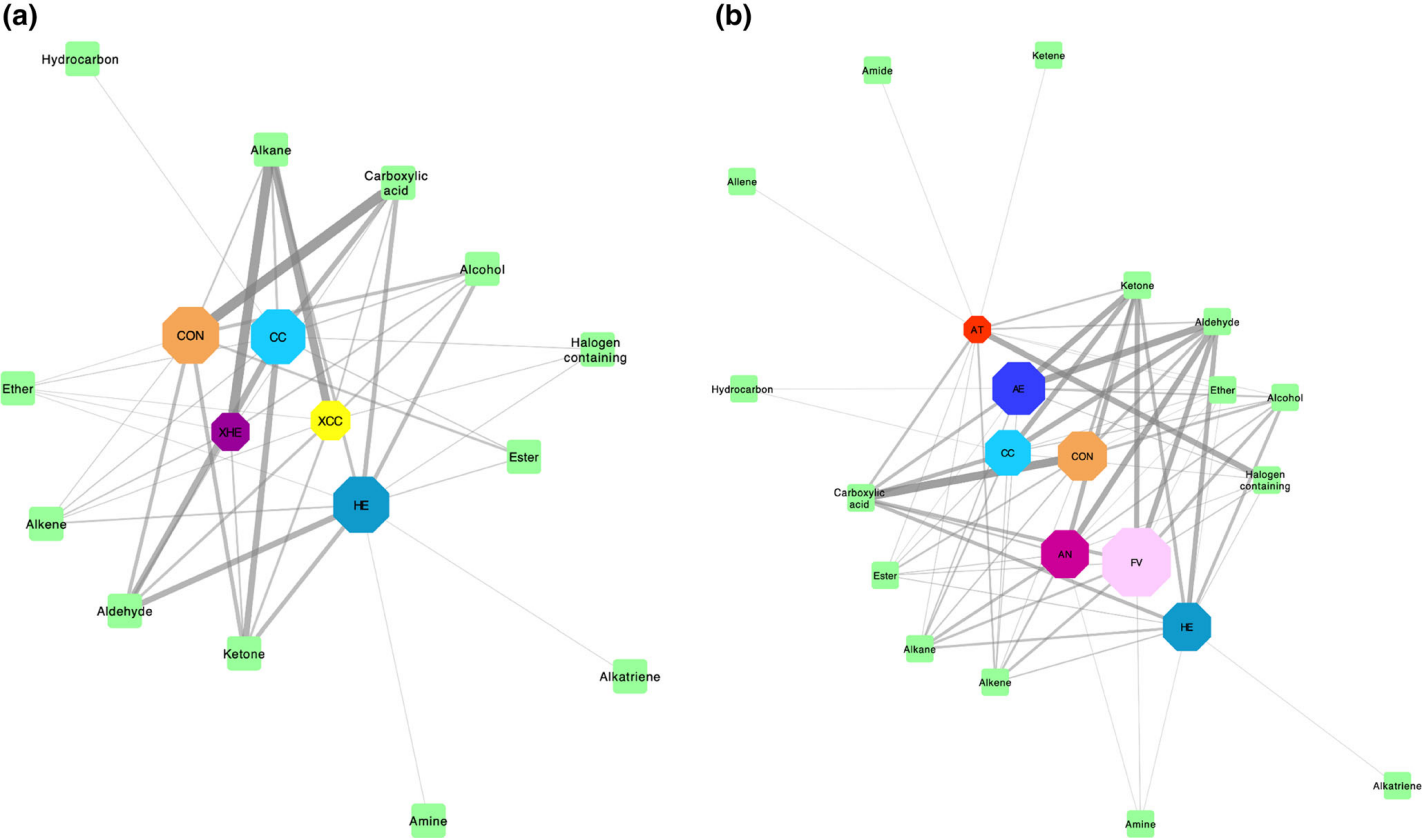
Bipartite network of substrates (hexagons) and compounds (squares) found within them. Substrate node size represents the total number of compounds detected. Edge width indicates the quantity of a particular compound found within a connected substrate. (a) Seaweed extracts and their respective whole seaweed samples against the positive (AT) and negative (CON) controls (CC, *Chondrus crispus*; HE, *Himanthalia elongata*; XCC: *Chondrus crispus* extract; XHE, *Himanthalia elongata* extract; AT, *Asparagopsis taxiformis*; CON, grass silage). (b) Whole seaweed samples tested and grass silage (AE, *Alaria esculenta*; CC, *Chondrus crispus*; FV, *Fucus vesiculosus*; HE, *Himanthalia elongata*; AN, *Ascophyllum nodosum*; AT, *Asparagopsis taxiformis*; CON, grass silage).

**Figure 2:**
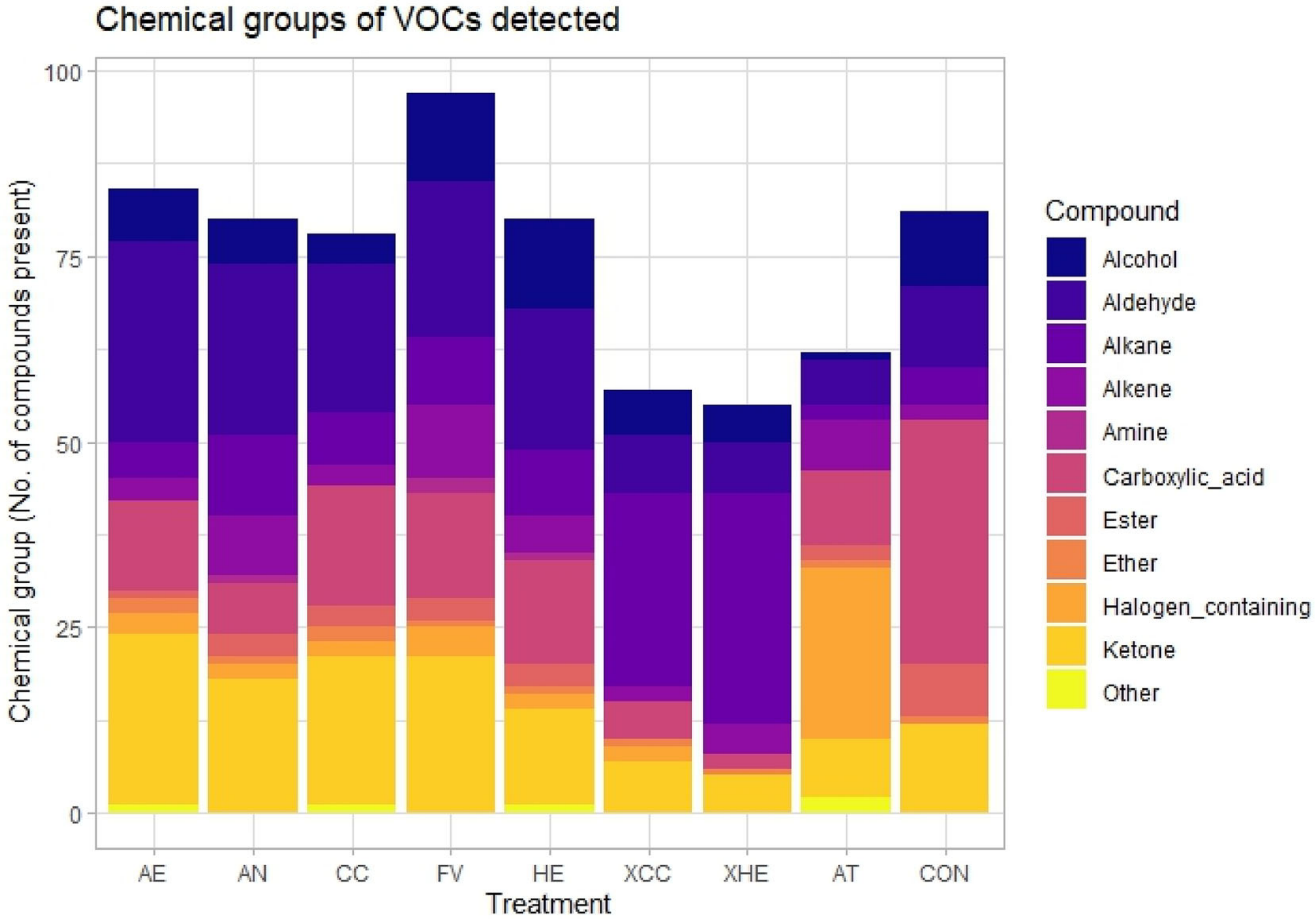
The quantity of each volatile organic chemical group detected in each test substrate following solid‐phase micro‐extraction analysis. AE, *Alaria esculenta*; AN, *Ascophyllum nodosum*; CC, *Chondrus crispus*; FV, *Fucus vesiculosus*; HE, *Himanthalia elongata*; XCC, *Chondrus crispus* extract; XHE, *Himanthalia elongata* extract; AT, *Asparagopsis taxiformis*; CON, grass silage. See Supporting Information S1 for exact values.

The XHE, XCC and AT treatments had a lower number of total VOCs present in comparison to the whole seaweed samples and grass silage (CON) treatments (Figs 1(a),(b) and 2; Table 3). The greatest amount of VOC overlap with CON was seen with the five whole seaweeds investigated, whereas the XCC and XHE extracts were similar to one another but different from the AT positive control. The whole seaweeds CC and HE have a numerically higher VOC content in comparison to their extracts XCC and XHE, with a difference of 21 and 25, respectively (Figs 1(a) and 2). The XCC and XHE treatments had a greater quantity of alkane compounds compared to all other treatments (Fig. 2). The CON treatment had the highest quantity of carboxylic acid compounds (33), while AT had the highest presence of halogenated compounds (23) (Fig. 2; Table 3) and unique compounds (Fig. 1(b)). Tribromomethane (CHBr_3_) was present in all substrates except for CON and XHE, while 1‐bromooctane was the only other CHBr_3_‐containing compound detected in species other than AT (Table 3). See Supporting Information S1 for exact values.

**Table 3:** List of halogenated compounds detected in tested samples in the SPME analysis (✓ denotes presence)

ID	AT	CON	HE	FV	AE	CC	XHE	XCC	AN
2‐Propanol, 1,3‐dibromo‐	✓								
2‐Propanone, 1,1,3,3‐tetrachloro‐	✓								
2‐Propanone, 1,1‐dichloro‐	✓								
2‐Propanone, 1,3‐dichloro‐	✓								
2‐Propanone, 1‐chloro‐	✓								
2‐Propenoic acid, 3,3‐dibromo‐, methyl ester	✓								
2‐Propenoic acid, 3‐chloro‐, (*Z*)‐	✓								
Acetaldehyde, chloro‐	✓								
Acetic acid, bromoiodo‐, methyl ester	✓								
Benzene, (bromomethyl)‐	✓								
Benzyl chloride	✓			✓	✓	✓			
Carbon tetrabromide	✓								
Chloromethane	✓								
Decane, 1‐bromo‐	✓								
Ethane, iodo‐			✓		✓				
Methane, bromo‐	✓								
Methane, bromodiiodo‐	✓								
Methane, dibromo‐	✓								
Methane, dibromochloro‐	✓								
Methane, dichloronitro‐	✓								
Methane, iodo‐	✓								
Methane, tribromo‐	✓		✓	✓	✓	✓		✓	✓
Methane, triiodo‐	✓								
Octane, 1‐bromo‐	✓			✓					✓
Octane, 1‐chloro‐				✓					
Propane, 1‐chloro‐2‐methyl‐								✓	
Total	23	0	2	4	3	2	0	2	2

AT, *Asparagopsis taxiformis*; CON, grass silage; HE, *Himanthalia elongata*; FV, *Fucus vesiculosus*; AE, *Alaria esculenta*; CC, *Chondrus crispus*; *X*HE, *Himanthalia elongata* extract; XCC, *Chondrus crispus* extract; AN, *Ascophyllum nodosum*.

### *In vitro* rumen fermentation

**Figure 3:**
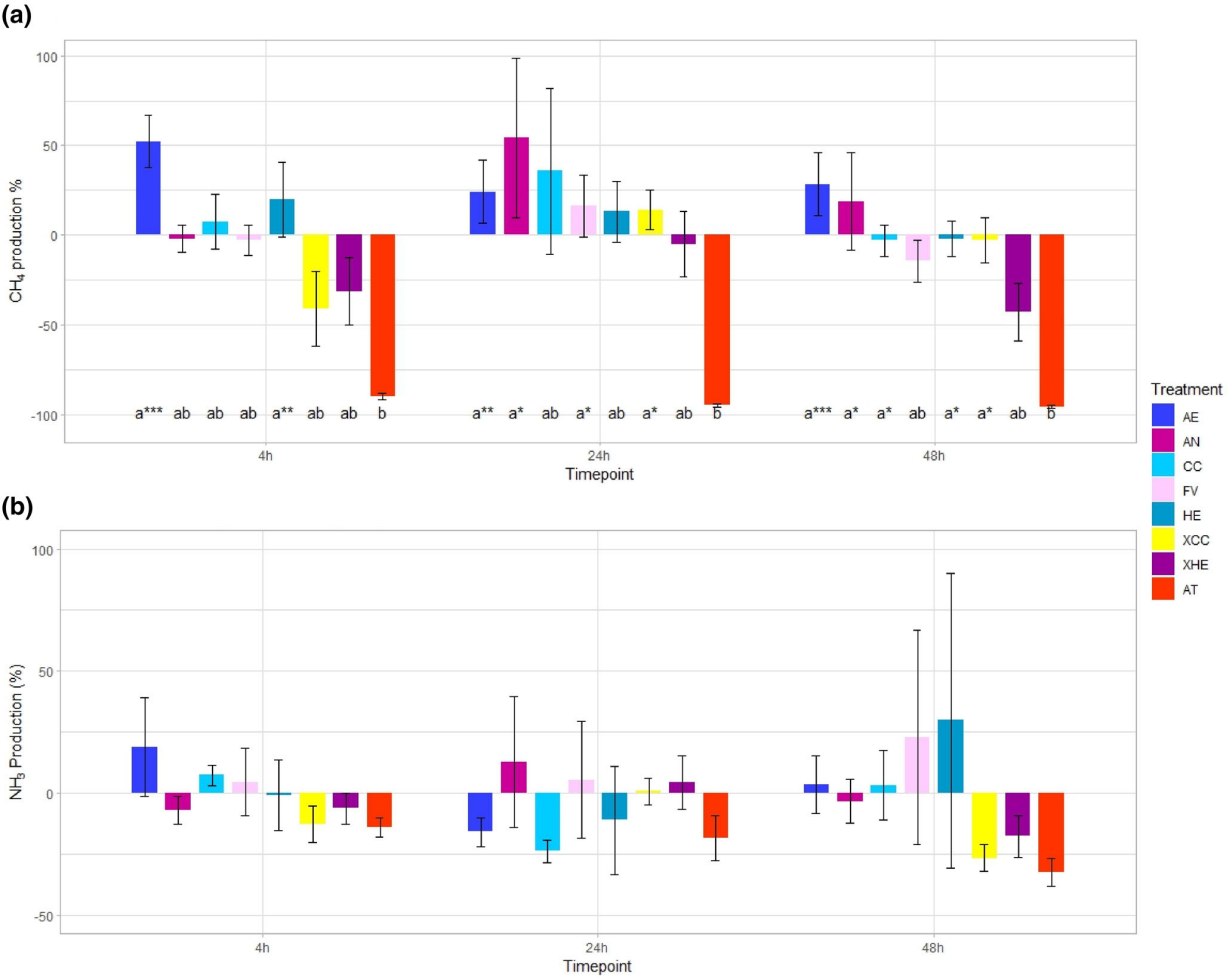
The effect of 4% inclusion of seaweed substrate across three timepoints expressed as percent change from the negative control on (a) CH_4_ production and (b) NH_3_ production. AE, *Alaria esculenta*; AN, *Ascophyllum nodosum*; CC, *Chondrus crispus*; FV, *Fucus vesiculosus*; HE, *Himanthalia elongata*; XCC, *Chondrus crispus* extract; *X*HE, *Himanthalia elongata* extract; AT, *Asparagopsis taxiformis*. Asterisks indicate interactions across treatments within timepoints (*P < 0.05; **P < 0.01; ***P < 0.001) and error bars represent the standard error of the mean.

AT consistently reduced CH_4_ production across timepoints in comparison to the respective negative grass silage control (~93.3%) and other tested seaweeds. At the 4 h timepoint AT was significantly different from AE (*P* < 0.001) and HE (*P* < 0.01). AN, *Fucus vesiculosus* (FV), XCC and XHE elicited a reduction in CH_4_ production in comparison to the negative controls, showing 2.0%, 3.0%, 40.9% and 31.1% reductions, respectively. At the 24 h timepoint AT was significantly different from AE (*P* < 0.01), AN (*P* < 0.05), FV (*P* < 0.05) and XCC (*P* < 0.05) but not from CC, HE or XHE (Fig. 3). XHE was the only tested seaweed to cause a reduction in CH_4_ production (4.9%) at the 24 h timepoint. High variability in standard error of the mean (SEM) was observed for AN (±44.39) and CC (±46.33) at the 24 h timepoint in comparison to all other treatments, likely due to variation in animal responses. At the 48 h timepoint AT was significantly different from AE (*P* < 0.001), AN, (*P* < 0.05), HE (*P* < 0.05) and XCC (*P* < 0.05), while FV (14.4%), CC (2.9%), HE (1.9%), XCC (2.8%) and XHE (42.8%) all showed reductions in CH_4_ production over the negative control at the 48 h timepoint.

No significant differences were observed within timepoints across treatments for NH_3_‐N production (Fig. 3). XCC, XHE and AT had the most consistent responses across timepoints, with reductions of 12.7%, 6.3% and 13.9%, respectively, at the 4 h timepoint, +0.8%, +4.5%, −18.4% at the 24 h timepoint, and − 26.5%, −17.6% and −32.4% at the 48 h timepoint. Unexpectedly, due to the high PT content, the greatest SEM was observed for FV (±43.82) and HE (±60.36) at the 48 h timepoint. The pH results at 0, 4, 24 and 48 h timepoints are displayed in Table 4. No significant differences in pH within timepoint and across treatments were recorded for the 0, 4 and 24 h timepoints. At the 48 h timepoint, CC was significantly different from XHE (P < 0.05).

**Table 4:** The average pH values recorded for each treatment (*n* = 6) at each timepoint, and standard error of the mean (SEM) and *P*‐value across treatments at each timepoint

pH	Whole seaweed							Seaweed extracts		SEM	*P*‐value
	CON	AE	AN	AT	CC	FV	HE	XCC	XHE		
0 h	6.63	6.64	6.70	6.67	6.62	6.69	6.68	6.75	6.62	0.015	0.903
4 h	6.54	6.64	6.52	6.54	6.61	6.67	6.66	6.61	6.54	0.044	0.198
24 h	5.88	5.81	5.78	5.95	5.91	5.99	5.90	5.78	5.87	0.123	0.487
48 h	5.50	5.78	5.58	5.68	5.83b	5.70	5.66	5.26	5.27a	0.131	0.025

Letters after entries indicate significant differences within timepoints across treatments (*P* < 0.05). CON, grass silage; AE, *Alaria esculenta*; AN, *Ascophyllum nodosum*; AT, *Asparagopsis taxiformis*; CC, *Chondrus crispus*; FV, *Fucus vesiculosus*; HE, *Himanthalia elongata*; XCC, *Chondrus crispus* extract; *X*HE, *Himanthalia elongata* extract.

**Figure 4:**
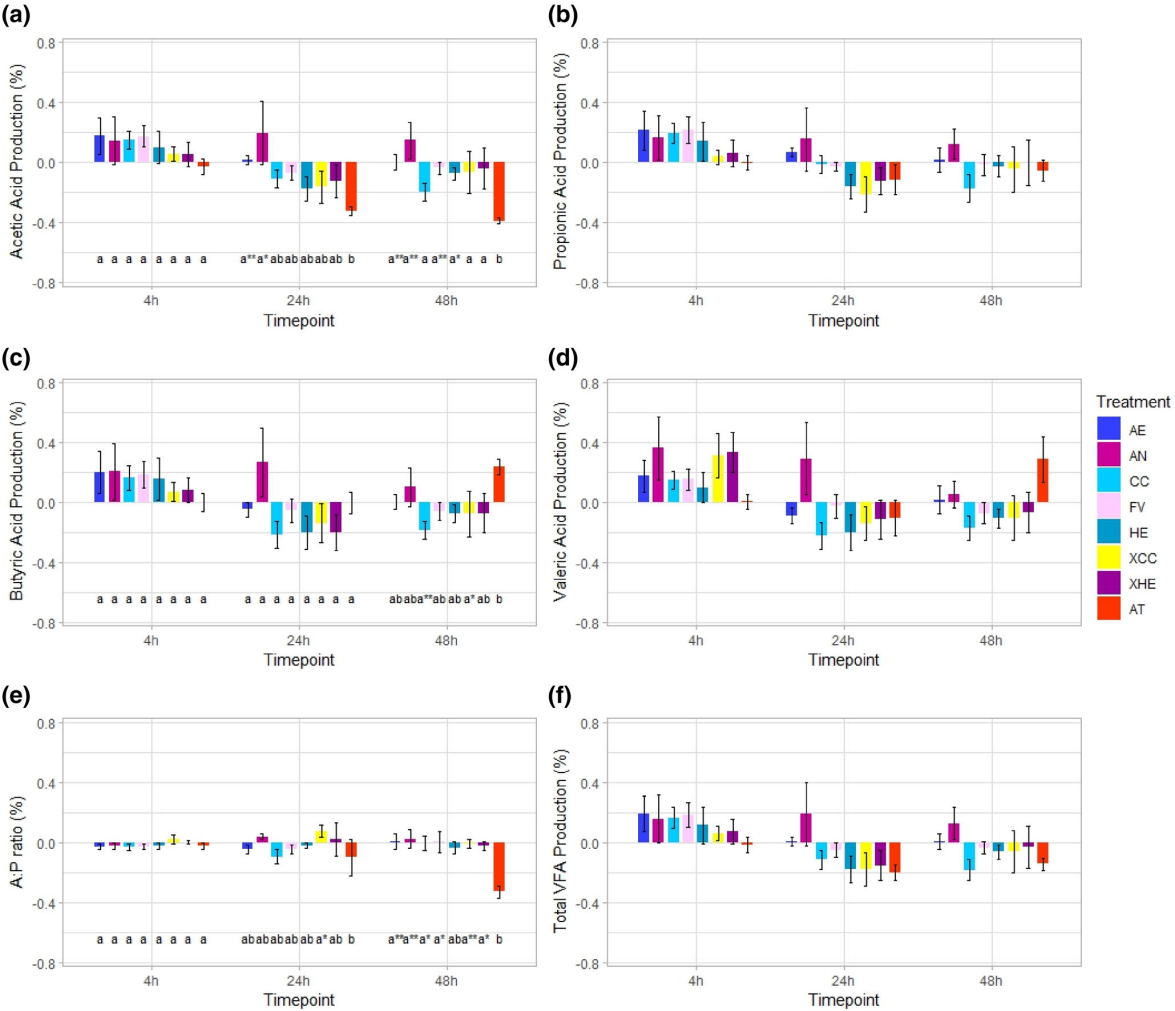
The effect of 4% inclusion of seaweed substrate across three timepoints expressed as percent change from the negative control on (a) acetic acid production, (b) propionic acid production, (c) butyric acid production, (d) valeric Acid production, (e) acetic acid:propionic acid ratio and (f) total VFA production. AE, *Alaria esculenta*; AN, *Ascophyllum nodosum*; CC, *Chondrus crispus*; FV, *Fucus vesiculosus*; HE, *Himanthalia elongata*; XCC, *Chondrus crispus* extract; *X*HE, *Himanthalia elongata* extract; AT, *Asparagopsis taxiformis*. Asterisks indicate interactions across treatments within timepoints (*P < 0.05; **P < 0.01; ***P < 0.001). Error bars represent the standard error of the mean.

No significant differences were observed in propionic acid, valeric acid or total VFA production across treatments at different timepoints (*P* > 0.05; Fig. 4(b),(d),(f)). However, for acetic acid production, AT was significantly different from AE (*P* < 0.01) and AN (*P* < 0.05) at the 24 h timepoint and from all treatments at the 48 h timepoint (*P* < 0.05; Fig. 4(a)). This relates to the significant difference observed for the acetic acid:propionic acid ratio for AT from all other treatments (*P* < 0.05), apart from HE at the 48 h timepoint (Fig. 4(e)). For butyric acid, AT was significantly different from CC (*P* < 0.01) and XCC (*P* < 0.05) at the 48 h timepoint, having higher production in respect to the negative control (Fig. 4(c)). AN did not follow the same trend as the other tested seaweeds, having numerically higher acetic, propionic, butyric and valeric acid production in comparison to its respective negative controls.

## DISCUSSION

This study aims to identify temperate seaweeds with a decreased carbon footprint in terms of distribution distance to countries with an aligned climate, with strong anti‐methanogenic characteristics that could potentially be brought forward to animal studies at realistic farm‐level inclusion rates. Of the temperate seaweeds tested, the results indicate that the greatest opportunity for development of a CH_4_‐mitigating feed ingredient exists for *F. vesiculosus, H. elongata* and *H. elongata* extract. The secondary interest was to assess whether any of the investigated species could provide the dual benefit of reducing enteric NH_3_‐N alongside CH_4_ without negatively impacting VFA production. The two seaweed extracts (XHE, XCC) had the most consistent reduction in NH_3_‐N across timepoints (4, 24, 48 h). Except for XHE, the high PT‐containing species (FV, HE) did not elicit the expected reductions to NH_3_‐N and no significant differences were observed across the tested species.

In the current study, five temperate seaweeds and two seaweed extracts were assessed with differing bioactive compounds and thus varying modes of action on the rumen environment; the PT containing *Alaria esculenta, Ascophyllum nodosum, F. vesiculosus, H. elongata* and *H. elongata* extract; the flavonoid containing *C. crispus* and *C. crispus* extract against the bromoform containing *Asparagopsis taxiformis* positive control. All the tested seaweeds produced variable responses on CH_4_ and NH_3_‐N production *in vitro*, with reduced variability observed for VFA production. The results from the present study indicated that the selected 4% inclusion rate was below the threshold to elicit a consistent response across timepoints, and thus, while the PT content was high in some species (HE and XHE), the total dietary inclusion was low.

### Effects of polyphenol‐containing seaweed species on methane production

The higher‐than‐expected SEM across six replicates per treatment and timepoint suggests that animal and rumen microbiome variations impact seaweeds' effect on emissions over 48 h. The variation observed could have been reduced from mixing the rumen fluid contents from the six cows to make one homogeneous mix. The largest range in SEM was observed for two PT containing species AE and AN and the flavonoid‐containing red seaweed CC, being particularly notable at 24 h. Based on existing literature and the inclusion rate used, increases in CH_4_ were unexpected at the 24 h for AE, AN and CC and 48 h timepoints for AE and AN. To date, there are no published papers using whole AE: however, Ramin *et al*.^45^ explored the effects of three AE extracts and found a dose‐dependent response, with reductions ranging from 0.5% to 16.5% at the highest inclusion rate. Interestingly, all three extracts had similar polyphenol content (5.3, 5.1, 5.0 g kg^−1^ DM) but the effect on CH_4_ differed; PT content was not reported. In the present study the AE tested had a lower polyphenolic content than the Ramin *et al*.^45^ study, which could explain the difference in response. For AN, Pandey *et al*.^46^ used a 20% inclusion rate with a maize silage substrate attaining a 34.5% CH_4_ reduction at the 48 h timepoint. Künzel *et al*.^47^ used a 5% inclusion rate and found an 8.9% CH_4_ concentration reduction during days 7–13 of a RUSITEC (rumen simulation technique)‐based fermentation. Roskam *et al*.^35^ found a 3.73% increase in CH_4_ using a 1% inclusion rate on days 15–21 of a RUSITEC experiment. For CC, a previous study found CH₄ reductions of 8.4% at 6% inclusion and 13.4% *in vivo*.^48^ A 20% inclusion (maize silage) reduced CH₄ by 56.5% at 48 h,^45^ while a 0.5% inclusion increased CH₄ by ~7% at 24 h.^49^ Previous research has indicated that flavonoids have a wide range of biological activity, which includes anti‐methanogenic due to the direct effect on the rumen methanogenic archaea.^50^ This suggests that CC and the particular flavonoid compounds that it contains should be investigated further to explore the mode of action and quantity required to elicit a response.

The variation in SEM observed could be the result of animal genetics and physiology, rumen microbiome community, water consumption and dietary changes. Goopy *et al*.^51^ found ruminal methanogenesis in sheep to be heritable, with lower CH₄ output linked to smaller rumens, reduced fermentation and altered nutrient flux. Daily variations in saliva production may also influence the rumen microbiome.^52^ Moreover, HE and FV had the highest PT content among the brown seaweeds studied (5.53 and 3.35 μL mL^−1^ g^−1^, respectively), resulting in a more consistent and pronounced CH₄ response. The CH_4_ production from HE decreased over the three investigated timepoints, with a reduction of 2.0% being recorded at the 48 h timepoint. Pandey *et al*.^46^ explored the effects of harvesting season on *in vitro* rumen CH_4_ production for HE, and found that when incubated with maize silage the spring harvested had ~2× the mitigating potential over the autumn HE (15% *vs*. 6% CH_4_ reduction as percent of total gas production). Interestingly, the total polyphenol content was 29% lower in the spring‐harvested HE (spring = 26.9 ± 0.45, autumn = 38.1 ± 1.18 mg PGE g^−1^ DM). In the current study, XHE had a significantly higher PT content and elicited a greater CH_4_ reduction compared to the whole HE.

Similarly, at 48 h, FV reduced CH₄ by 14.4%, though variations exist across studies, such as a 3.6% reduction during days 7–14 of a RUSITEC study,^47^ ~1% reduction at 0.5% organic matter inclusion at 24 h^49^ and reductions of 63% per gram OM when incubated with corn silage at an inclusion level of 20%.^46^ Interestingly, when investigated *in vivo* in dairy cows FV did not elicit a mitigating effect on CH_4_ production, likely due to the reduced inclusion rate of 4% and difference in basal diet.^53^ Like HE, Pandey *et al*.^46^ found that the polyphenol content was lower in the spring‐harvested FV, while the CH_4_ reduction was greater for the autumn harvested FV. It should be noted that other polyphenolic compounds could be influencing the response observed; they were not quantified in the present study, the main focus being on PT content.

### Effect of bromoform containing seaweed *spp.* on methane production

AT had a consistently lower SEM range across timepoints, having the ability to elicit an effect immediately and overcome the challenges presented from the variation in rumen fluid. Consistent with existing literature, AT significantly reduced CH_4_ at all timepoints over CON (85–95% reduction) and in comparison to the other tested seaweeds.^16^, ^35^, ^54^ Additionally, Roque *et al*.^55^ used a comparable inclusion rate of 5% (organic matter) of AT and found reductions of 95% *in vitro*. The authors also explored AT provision *in vitro* at inclusions < 1%, with equally positive responses attained.^16^ The anti‐methanogenic effect observed from red seaweed provision is linked to the presence of halogenated compounds and, notably, bromoform. Across red seaweed species, their ability to bioaccumulate halogenated compounds in their tissues differs.^56^ AT and *A. armata* continually show high concentrations of bromoform at 1–5% of dry mass^57^ and have a consistent response on rumen enteric CH_4_ production *in vitro*.^16^, ^35^, ^54^ Reyes *et al*.^48^ also showed that CC possesses the enzymes required to synthesise halogenated compounds; however, none of the samples they tested had detectable levels of bromoform or brominated metabolites. The authors suggested that CC contains unknown bioactive compounds that can interfere with methanogenesis. In support of Reyes *et al*.,^48^ the present study identified only two halogen‐containing volatile compounds in CC, while the high polyphenolic content suggests that flavonoids could be responsible for the response observed.

### Effects of seaweed extraction on chemical composition

The extraction process increased the PT content in HE and transformed other compounds while positively affecting the ability of HE and CC to reduce CH_4_ *in vitro*. Differences were observed in the VOC content of XHE and HE (total respective VOCs of 80 and 55), with whole HE having a greater quantity of alcohols, aldehydes and carboxylic acid groups and fewer alkanes over XHE. The exothermic extraction process could remove several chemical compounds while also transforming others, potentially removing noise around the PTs in brown seaweeds and allowing faster activity within the rumen environment or creating new bioactive compounds that can act on methanogenesis, further emphasising the need to explore the chemical composition of seaweeds and the modes of action in the rumen environment. It is important to note that, within seaweed species, harvesting conditions (i.e., season, geographical location) could lead to different results due to changes in bioactive concentrations.^58^

### Effects of seaweed provision on rumen ammonia production

The authors hypothesised that the presence of PTs in the tested brown seaweeds would provoke a response on ruminal NH_3_‐N production, especially those with the highest PT content. This is due to the NH_3_‐H reductions observed from CT provision in ruminants.^26^, ^27^ FV, HE and XHE contain the largest amount of PTs of the tested seaweeds. FV reduced NH_3_‐N at the 24 h timepoint but not at the 4 or 48 h timepoints; HE increased ruminal NH_3_‐N across all timepoints, with large variations in results observed at the 24 and 48 h timepoints; XHE reduced NH_3_‐N at the 4 and 48 h timepoints but not 24 h. PTs have been reported to exhibit effects similar to terrestrial plant tannins, binding to both protein and fibre, with the highest affinity for proteins.^59^, ^60^ The formation of tannin–protein complexes in the rumen and subsequent pH‐driven dissociation in the abomasum increases the amount of by‐pass protein and reduces the amount of ruminal NH_3_‐N.^61^, ^62^, ^63^ Wang *et al*.^26^ showed a dose‐dependent response after a 48 h rumen *in vitro* incubation on NH_3_‐N concentration when PTs extracted from AN were provided alongside a mixed forage substrate. The highest concentration of 500 mg mL^−1^ reduced NH_3_‐N by 49.4% and the lowest concentration of 125 mg mL^−1^ reduced NH_3_‐N by 10.4%.^26^ In the current study, the amount of PT provided by the same species was 2.21 mg mL^−1^ g^−1^ and the decrease in NH_3_‐N was 3.3% at the comparable 48 h timepoint, while an increase of 12.8% NH_3_‐N was observed at the 24 h timepoint, showing that the lower PT inclusion resulted in higher variability and thus a 4% inclusion rate of AN is too low to cause a significant positive response on NH_3_‐N production. Kim *et al*.^64^ suggested that PTs could be degraded into phloroglucinol units (monomers of PTs), which can be broken down into VFAs.^65^, ^66^ In the present study, it is plausible that the PTs were broken down at different rates dependent on PT structure and thus have lower binding affinity than expected. Contrastingly, the high PT containing XHE resulted in a decrease in NH_3_‐N at the 4 h (6.3%) and 48 h (17.6%) timepoints. However, reductions in NH_3_‐N were observed for CC and XCC across timepoints, highlighting the need to explore the other bioactive compounds present in seaweeds and how they interact with the rumen microbiome. This further suggests that the effect on rumen NH_3_‐N is not solely from PT provision.

### Effect of seaweed provision on rumen pH and VFA production

The link between PT protein binding and pH must also be considered. The supplementation of each brown whole seaweed (AE, AN, FV, HE) marginally increased the pH at 48 h of incubation, which may be attributable to PTs' rumen fermentation‐impairing property.^26^, ^58^, ^62^ Tannin's ability to bind proteins increases with increasing pH within the range pH 3.0–7.0.^67^ The pH was below the desirable range of pH 6.5–pH 7.0 for all treatments and the negative control at the 24 and 48 h timepoints, which could have impacted the affinity of PT–protein complex formation. The average starting pH (0 h) ranged from pH 6.62 to 6.75, suggesting that the fermentation within the closed *in vitro* system caused a reduction in pH over time. Thus, to fully assess the impact of the treatments, the study should be repeated in a continuous *in vitro* system (e.g., RUSITEC).

Surprisingly, there was not an increase in propionate production when AT was provided, but there was an increase in butyric acid and valeric acid production (and other hydrogen sinks) and a decrease in acetic acid production, which ultimately led to a more favourable A:P ratio. AT has been shown to have an antimicrobial effect on the rumen environment due to the presence of halogenated compounds, which likely induce shifts in rumen microbiota and consequently alter rumen fermentation patterns.^68^ In addition, reductions in enteric CH_4_ have been connected to a significant shift in the A:P ratio.^16^, ^69^ While the tested seaweeds (aside from AN) showed a reduction in acetic acid production at the 24 and 48 h timepoints, the lack of increase in the other reported VFAs suggests that their provision limits rumen fermentation and thus would have a negative impact on animal performance. However, for total VFA production, AE, FV and XHE had the least notable difference from their respective negative controls, suggesting that the impact would be minimal. AN increased all VFAs and CH_4_ production, suggesting an increased rate of rumen fermentation and methanogenesis through its provision in this particular study.

## CONCLUSIONS

The present study has provided a novel insight into the use of temperate seaweeds in ruminant diets and the effects on rumen CH_4_, NH_3_‐N, VFA production and pH at a viable dietary inclusion level for *in vivo* provision. The study has provided scope for further investigation of bioactive compounds due to changes in rumen fermentation outputs observed from CC provision that does not contain high quantities of PTs or halogenated compounds but instead includes flavonoid compounds alongside the differences in VOCs across species, while the known high presence of halogenated compounds found in AT allows it to stand alone in its ability to mitigate CH_4_ production. The study further highlights the variability in response from whole non‐halogen‐containing seaweed species with harvesting season adding complexity to result consistency. Notably, the results from the present study indicate the greatest opportunity for FV, HE and XHE of the temperate seaweeds tested for CH_4_ mitigation in ruminants. This study is the first of its kind to investigate these species with a grass silage base substrate *in vitro*, and HE and XHE are yet to be researched as dietary feed ingredients in ruminant *in vivo* studies.

## FUNDING INFORMATION

This work was supported by the 'SEASOLUTIONS’ European project, European Union’s Horizon 2020 (grant number 696356) and the Professor John Glover Memorial Award 2024 and the Department for the Economy in Northern Ireland (DfE).

## Supporting information


**Supporting Information S1:** The quantity of each chemical group detected in each test substrate following SPME analysis. CON, grass silage; AE, *Alaria esculenta*; AN, *Ascophyllum nodosum*; CC, *Chondrus crispus*; FV, *Fucus vesiculosus*; HE, *Himanthalia elongata*; XCC, *Chondrus crispus*; XHE, *Himanthalia elongata*; AT, *Asparagopsis taxiformis*.

## Data Availability

The data that support the findings of this study are available from the corresponding author upon reasonable request.
