# Supplementary material for: Temperate seaweeds Himanthalia elongata and Fucus vesiculosus significantly reduce rumen methane emissions in vitro due to their high phlorotannin content
Source: J Sci Food Agric. 2025 Jul 2;105(13):7522–34. doi: 10.1002/jsfa.70016 (PMC12439089; doi:10.1002/jsfa.70016)
Supplement: Supplementary file 1 — Supporting Information S1: The quantity of each chemical group detected in each test substrate following SPME analysis. CON, grass silage; AE, Alaria esculenta; AN, Ascophyllum nodosum; CC, Chondrus crispus; FV, Fucus vesiculosus; HE, Himanthalia elongata; XCC, Chondrus crispus; XHE, Himanthalia elongata; AT, Asparagopsis taxiformis. [file JSFA-105-7522-s001.docx]

**Supplementary materials**

S1: The quantity of each chemical group detected in each test substrate following SPME analysis. CON: grass silage, AE: *Alaria esculenta,* AN: *Ascophyllum nodosum,* CC: *Chondrus crispus,* FV: *Fucus vesiculosus,* HE: *Himanthalia elongata,* XCC: *Chondrus crispus, X*HE: *Himanthalia elongata,* AT: *Asparagopsis taxiformis*.

| **Chemical group** | **CON** | **AE** | **AN** | **CC** | **FV** | **HE** | **XCC** | **XHE** | **AT** |
| --- | --- | --- | --- | --- | --- | --- | --- | --- | --- |
| Alcohol | 10 | 7 | 6 | 4 | 12 | 12 | 6 | 5 | 1 |
| Aldehyde | 11 | 27 | 23 | 20 | 21 | 19 | 8 | 7 | 6 |
| Alkane | 5 | 5 | 11 | 7 | 9 | 9 | 26 | 31 | 2 |
| Alkene | 2 | 3 | 8 | 3 | 10 | 5 | 2 | 4 | 7 |
| Amine | 0 | 0 | 1 | 0 | 2 | 1 | 0 | 0 | 0 |
| Carboxylic acid | 33 | 12 | 7 | 16 | 14 | 14 | 5 | 2 | 10 |
| Ester | 7 | 1 | 3 | 3 | 3 | 3 | 0 | 0 | 2 |
| Ether | 1 | 2 | 1 | 2 | 1 | 1 | 1 | 1 | 1 |
| Halogen containing | 0 | 3 | 2 | 2 | 4 | 2 | 2 | 0 | 23 |
| Ketone | 12 | 23 | 18 | 20 | 21 | 13 | 7 | 5 | 8 |
| Other | 0 | 1 | 0 | 1 | 0 | 1 | 0 | 0 | 2 |
|  | | | | | | | | | |
| Total | 81 | 84 | 80 | 78 | 97 | 80 | 57 | 55 | 62 |
